# Impact of medical checkup parameters on major adverse cardiovascular events in the general Japanese population

**DOI:** 10.1016/j.pmedr.2024.102600

**Published:** 2024-01-06

**Authors:** Tomonori Sugiura, Hiroyuki Takase, Yasuaki Dohi, Sumiyo Yamashita, Yoshihiro Seo

**Affiliations:** a Department of Cardiology, Nagoya City University Graduate School of Medical Sciences, 1 Kawasumi, Mizuho-cho, Mizuho-ku, Nagoya 467-8601, Japan; b Department of Internal Medicine, Enshu Hospital, 1-1-1 Chuo, Chuo-ku, Hamamatsu 430-0929, Japan; c Department of Internal Medicine, Faculty of Rehabilitation Sciences, Nagoya Gakuin University, 1-25 Atsuta Nishi-machi, Atsuta-ku, Nagoya 456-8612, Japan; d Department of Cardiology, Nagoya City University Mirai Kousei Hospital, 2-1501 Sekobo, Meito-ku, Nagoya 465-8650, Japan

**Keywords:** B-type natriuretic peptide (BNP), Medical checkup parameters, Predictor, Cardiovascular events, General population, Metabolic syndrome

## Abstract

Medical checkups play a role in the identification of individuals at increased cardiovascular risk. However, the impact of each medical examination parameter on the incidence of major adverse cardiovascular events (MACE) has not been intensively studied. Here we assessed the predictors of MACE among parameters examined during medical checkups in the general Japanese population. A total of 13,522 individuals (mean age, 52.8 ± 12.3 years) who participated in our medical checkup program from 2008 to 2015 were followed up for a median of 1,827 days with the endpoint of MACE. MACE included cardiovascular death, non-fatal myocardial infarction, angina, decompensated heart failure, stroke, and other cardiovascular events requiring hospitalization. Possible associations between MACE and baseline clinical test parameters were investigated. During follow-up, MACE occurred in 196 participants. Participants with hypertension, diabetes mellitus, dyslipidemia, or metabolic syndrome were at increased risk of MACE on the univariate analysis. Multivariate Cox hazard analysis demonstrated that male sex, age, systolic blood pressure, and baseline B-type natriuretic peptide level were independently correlated with future MACE after the adjustment for confounders; the impact of B-type natriuretic peptide was most prominent among the investigated variables. These results suggest that B-type natriuretic peptide level obtained during a medical checkup examination is an independent and strong predictor of MACE. The inclusion of BNP as part of medical checkup parameters may improve the ability to identify individuals at increased cardiovascular risk and prevent cardiovascular disease among them.

## 1 Introduction

In Japan, the number of patients with cardiovascular disease, such as coronary heart disease, heart failure, and stroke, is estimated to be 2.85 million (approximately 2 % of the Japanese population), while the cardiovascular mortality rate comprises 22.2 % of all deaths (The Ministry of Health, Labour and Welfare, 2022). Cardiovascular disease is a leading cause of death (World Health Organization, 2020), and seriously affects healthy life expectancy. Therefore, several policies that target the general population have been developed to reduce the morbidity and mortality of cardiovascular disease. Although numerous factors are involved in the development of cardiovascular disease, most are modifiable and lifestyle-related (Irie et al., 2006, Imano et al., 2011, Hayama-Terada et al., 2016, Li et al., 2021, Satoh et al., 2021). Medical checkups in the general population can help identify individual modifiable risk factors and improve health or prevent cardiovascular disease.

Medical checkups are routinely performed for most of the Japanese population annually. In 2019, about 74 % of men and 66 % of women in Japan underwent an annual/periodical medical checkup (The Ministry of Health, Labour and Welfare, 2019). These medical checkups primarily aim to prevent lifestyle-related diseases and major adverse cardiovascular events (MACE). A medical checkup examination usually includes several tests, such as anthropometry, blood pressure, glucose tolerance, and lipid profiling, that evaluate an individual’s risk of cardiovascular disease. Recently, B-type natriuretic peptide (BNP) has been highlighted as a predictor of cardiovascular disease (Di Angelantonio et al., 2009). However, most studies investigating the predictive value of BNP were conducted in patients with stable vascular disease or individuals at increased cardiovascular risk, and the clinical significance of relatively low BNP levels in the general population has not been established (Imano et al., 2011). Therefore, the present study was designed to investigate significant predictors of MACE among clinical tests performed during medical checkups including BNP.

## 2 Methods

### 2.1 Study design

The present cohort study recruited participants in our annual medical checkup program. This study was conducted in accordance with the Declaration of Helsinki and approved by the ethics committee of Enshu Hospital (approval number, 18–40). All participants provided written informed consent prior to the start of the study and at each follow-up visit.

### 2.2 Study participants and procedures

**Fig. 1:**
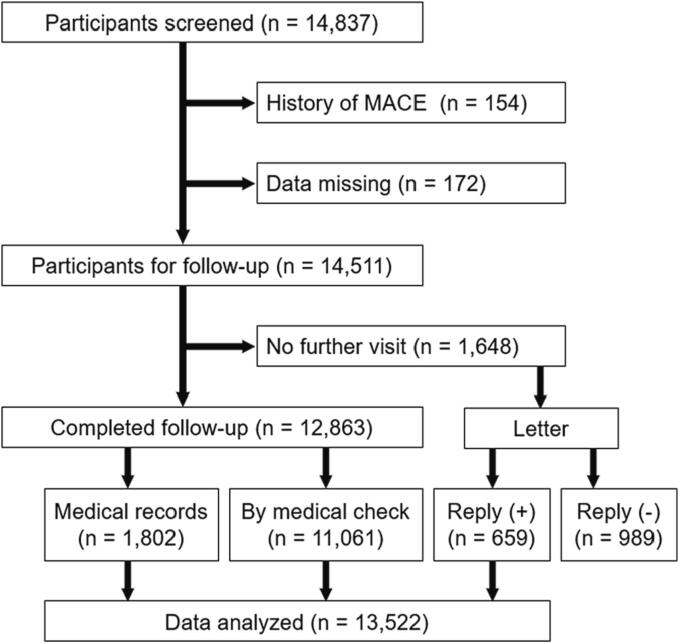
Flowchart of cumulative study participant exclusions, Hamamatsu, Japan, 2008–2016. Among 14,511 participants enrolled, 1,648 participants did not visit our hospital after the baseline examination. Major adverse cardiovascular events (MACE) were confirmed by medical records, medical checkup (a questionnaire followed by a history taking and medical examination by a doctor), or a letter.

Consecutive participants in our medical checkup program administered in 2008–2015 were given detailed information about the study protocol prior to the start of the medical checkup. Those who provided informed consent (*n* = 14,837) were then screened for eligibility. Participants with a history of MACE (*n* = 154) or for whom data were missing (*n* = 172) were excluded, while the remaining participants (*n* = 14,511) were included (Fig. 1). Our medical checkup program included an interview regarding health status, anthropometry, chest radiography, electrocardiography, and laboratory assessment of cardiovascular risk factors. The participants were followed up for a median 1,827 days (64,748 person–years), with the endpoint being the onset of MACE. The outcome was confirmed by medical record review; for participants whose medical records were not available, the presence or absence of MACE during follow-up was assessed by questionnaire followed by a history taking and medical examination by a doctor. Participants who reported MACE or a suspected MACE episode on the questionnaire consulted a doctor, who made a judgement about MACE status using a detailed history taking (including treatment regimen), medical examination, and electrocardiogram (ECG), chest radiography, and laboratory data. Furthermore, for participants who did not visit our hospital after the baseline examination, a letter was used to confirm MACE. Data obtained from participants who completed follow-up and those who responded to our letter asking about their health status were analyzed (Fig. 1). The possible association between MACE and baseline clinical test parameters including sex, age, waist circumference, blood pressure, kidney function, fasting plasma glucose, lipid profile, hemoglobin, BNP, ECG, smoking habit, and alcohol consumption were investigated. In a sub-analysis, participants taking medications that may affect the cardiovascular system were excluded, while the remaining participants were included (*n* = 10,475).

### 2.3 Definitions and clinical measurements

MACE was defined as cardiovascular death, non-fatal myocardial infarction, angina, decompensated heart failure, stroke, and other cardiovascular events (rapture of aortic aneurysm and acute aortic dissection). Myocardial infarction was defined based on typical symptoms, elevated cardiac enzymes (more than twice the upper limit of normal), ECG changes (ST segment elevation, ST segment depression, abnormal Q wave), asynergy of the left ventricular wall on echocardiography, and coronary angiographical findings. Angina was defined as ischemic coronary artery disease diagnosed by coronary angiography, computed tomography angiography, and/or cardiac scintigraphy requiring hospitalization for percutaneous transluminal coronary angioplasty or coronary artery bypass graft surgery. Decompensated heart failure was defined as heart failure requiring hospitalization. The definition of stroke was a sudden onset of focal and nonconvulsive neurological deficit lasting more than 24 h with typical brain imaging.

Diabetes mellitus was defined as a fasting plasma glucose ≥ 126 mg/dL, hemoglobin A1c (HbA1c) ≥ 6.5 % (The Committee of the Japan Diabetes Society on the Diagnostic Criteria of Diabetes Mellitus, 2010 (Seino et al., 2010)), or the use of antidiabetic medications. Dyslipidemia was defined as low-density lipoprotein cholesterol ≥ 140 mg/dL, high-density lipoprotein cholesterol < 40 mg/dL, triglycerides ≥ 150 mg/dL (Kinoshita et al., 2018), or the use of antidyslipidemic medications. Metabolic syndrome was defined based on the Japanese diagnostic criteria (waist circumference ≥ 85 cm for males and ≥ 90 cm for females and two or more of the following three criteria: (1) triglycerides ≥ 150 mg/dL and/or high-density lipoprotein cholesterol < 40 mg/dL, (2) systolic blood pressure ≥ 130 mmHg and/or diastolic blood pressure ≥ 85 mmHg, and (3) fasting plasma glucose ≥ 110 mg/dL) (Committee to Evaluate Diagnostic Standards for Metabolic Syndrome, 2005). Participants who had medication for each metabolic disorder were considered to meet each criterion. The estimated glomerular filtration rate (eGFR) was determined using the modified Modification of Diet in Renal Disease study formula for the Japanese population (Matsuo et al., 2009). Urinary protein was determined using a dipstick method (Arkray, Kyoto, Japan), which was interpreted by trained technicians and recorded as –, ±, 1+, 2+, 3+; proteinuria was defined as obtaining a result of 1+, 2+, or 3 +. Participants reported the frequency of alcohol consumption as ranging from 0 to 7 times/week, with frequent alcohol consumption defined as 6 or 7 times/week. For the measurement of BNP, 3 mL of blood was transferred to plastic tubes containing 4.5 mg of 2Na-ethylenediamine-tetraacetic acid. Plasma samples were prepared within 30 min using a pre-cooled centrifuge, immediately frozen, and stored at –70 °C until analysis. The plasma BNP concentration was determined using a commercially available chemiluminescence enzyme immunoassay (MI02 Shionogi BNP kit; Shionogi, Osaka, Japan) (Takase et al., 2011).

### 2.4 Statistical analysis

All analyses were performed using IBM SPSS Statistics 24 (IBM SPSS, Chicago, IL, USA). Data are presented as mean ± SD or as number and percentage of participants except for data on BNP and follow-up period, which are expressed as median and interquartile range. In addition, because the distribution of BNP was skewed to the right, log-transformed BNP values were used in the statistical analysis. Any significant difference between the means of two normally distributed data was determined using unpaired *t-*tests. The chi-squared test was used to compare categorical data. The Kaplan-Meier method was used to calculate the cumulative incidence rates of MACE.

To analyze the endpoint throughout the observation period, the significance of the difference in the cumulative incidence rates was evaluated by the log-rank test. The impact of medical examination parameters on the incidence of MACE was investigated using a Cox proportional hazard regression analysis. Model A was adjusted for male sex and age. Model B included variables that showed a significant correlation with MACE in Model A and other known risk factors as independent variables. Model C also included logBNP. All variables included in each model are listed in table footnotes. Hazard ratios (HRs) and 95 % confidence intervals (CIs) were calculated. In all cases, two-tailed tests were used, and *P* < 0.05 was considered statistically significant.

## 3 Results

The participants’ baseline characteristics are listed in Table 1. The mean value of each parameter in Table 1 was within the normal reference range. Participants taking medication for hypertension, diabetes mellitus, or dyslipidemia were 2,119 (60.4 % of those with hypertension), 712 (62.8 %), and 1,115 (19.7 %), respectively. After the baseline examination, the participants were followed up at the annual medical checkup, with MACE as the endpoint. The median follow-up period of the present study was 1,827 days (range, 105–3,181 days; 64,748 person–years). During the follow-up period, MACE occurred in 196 participants (Table 2). The outcome was confirmed using medical record review (*n* = 77), a questionnaire followed by a history taking and medical examination by a doctor at the medical checkup (*n* = 101), or a letter (*n* = 18). The dropout rate of the present study was 6.7 % (Fig. 1).

**Table 1:** Baseline characteristics of middle-aged study participants, Hamamatsu, Japan, 2008–2015 (Descriptive Statistics).

	Total (*n* = 13,522)
Sex; male, n (%)	8140 (60.2)
Age (years)	52.8 ± 12.3
Body mass index (kg/m^2^)	22.7 ± 3.3
Waist circumference (cm)	83.4 ± 9.1
Systolic blood pressure (mmHg)	123.6 ± 15.9
Diastolic blood pressure (mmHg)	75.9 ± 9.9
Pulse rate (bpm)	63.4 ± 9.4
Fasting plasma glucose (mg/dL)	96.7 ± 18.7
HbA1c (%)	5.68 ± 0.66
LDL-C (mg/dL)	120.4 ± 28.7
HDL-C (mg/dL)	60.0 ± 14.4
Triglyceride (mg/dL)	108.7 ± 77.2
Serum creatinine (mg/dL)	0.76 ± 0.19
eGFR (mL/min per 1.73 m^2^)	78.8 ± 14.6
Uric acid (mg/dL)	5.37 ± 1.39
Hemoglobin (g/dL)	14.0 ± 1.4
Sokolow-Lyon voltage (mV)	2.41 ± 0.79
BNP (pg/mL)	10.7 [6.4–17.8]
Current smoking status (%)	3,062 (22.6)
Frequent alcohol consumption (%)^A^	5,327 (39.4)
Proteinuria (%)	273 (2.0)
Hypertension (%)	3,511 (26.0)
Diabetes mellitus (%)	1,134 (8.4)
Dyslipidemia (%)	5,652 (41.8)
Metabolic syndrome (%)	1,681 (12.4)

Data are presented as mean ± SD, as *n* (%) or median [interquartile range].

^A^Frequent alcohol consumption was defined as the consumption of alcohol six or seven times per week.

Abbreviations: BNP, B-type natriuretic peptide; eGFR, estimated glomerular filtration rate; HbA1c, hemoglobin A1c; HDL-C, high-density lipoprotein cholesterol; LDL-C, low-density lipoprotein cholesterol.

**Table 2:** Major adverse cardiovascular events during the follow-up period in middle-aged study participants, Hamamatsu, Japan, 2008–2016.

	Total (*n* = 196)
Cardiovascular death	12
Non-fatal myocardial infarction	37
Angina	63
Decompensated heart failure	15
Stroke	66
Other cardiovascular events	3

Major adverse cardiovascular event is defined as cardiovascular death, non-fatal myocardial infarction, angina, decompensated heart failure, stroke, and other cardiovascular events (rapture of aortic aneurysm and acute aortic dissection).

### 3.1 Retrospective analysis of cardiovascular risk

A retrospective comparison of groups with and without MACE during the follow-up period revealed intergroup differences in baseline characteristics (Table 3). Risk factors such as age and blood pressure were more common in participants with versus without MACE, although the difference was not marked.

**Table 3:** Comparison of middle-aged study participants with and without major adverse cardiovascular event during follow-up (2008–2016), Hamamatsu, Japan: Retrospective analysis.

	without MACE (*n* = 13,326)	with MACE (*n* = 196)	*P* value^A^
Gender; male, n (%)	7,980 (59.9)	160 (81.6)	<0.001
Age (years)	52.7 ± 12.3	61.3 ± 8.9	<0.001
Body mass index (kg/m^2^)	22.7 ± 3.3	23.3 ± 2.9	0.008
Waist circumference (cm)	83.4 ± 9.1	86.4 ± 8.3	<0.001
Systolic blood pressure (mmHg)	123.4 ± 15.9	131.8 ± 15.7	<0.001
Diastolic blood pressure (mmHg)	75.8 ± 9.9	79.5 ± 10.3	<0.001
Pulse rate (bpm)	63.4 ± 9.4	62.4 ± 9.9	0.121
Fasting plasma glucose (mg/dL)	96.6 ± 18.5	101.1 ± 24.9	<0.001
HbA1c (%)	5.68 ± 0.66	5.87 ± 0.84	<0.001
LDL-C (mg/dL)	120.4 ± 28.7	120.5 ± 28.4	0.962
HDL-C (mg/dL)	60.1 ± 14.4	56.4 ± 14.1	<0.001
Triglyceride (mg/dL)	108.5 ± 77.3	118.7 ± 66.6	0.066
Serum creatinine (mg/dL)	0.76 ± 0.19	0.82 ± 0.21	<0.001
eGFR (mL/min per 1.73 m^2^)	78.9 ± 14.5	73.6 ± 15.0	<0.001
Uric acid (mg/dL)	5.37 ± 1.39	5.84 ± 1.22	<0.001
Hemoglobin (g/dL)	14.0 ± 1.5	14.1 ± 1.4	0.373
Sokolow-Lyon voltage (mV)	2.41 ± 0.79	2.73 ± 0.87	<0.001
BNP (pg/mL)	10.6 [6.3–17.7]	15.2 [9.5–28.8]	<0.001
Current smoking status (%)	3,015 (22.6)	47 (24.0)	0.716
Frequent alcohol consumption (%)^B^	5,240 (39.3)	87 (44.4)	0.172
Proteinuria (%)	271 (2.0)	2 (1.0)	0.456
Hypertension (%)	3,413 (25.6)	98 (50.0)	<0.001
Diabetes mellitus (%)	1,103 (8.3)	31 (15.8)	<0.001
Dyslipidemia (%)	5,555 (41.7)	97 (49.5)	0.034
Metabolic syndrome (%)	1,635 (12.3)	46 (23.5)	<0.001
Follow-up period (day)	1,828 [1,061–2,553]	1,230 [699–1,888]	<0.001

Data are presented as mean ± SD, as *n* (%) or median [interquartile range].

^A^Comparison between “without MACE” and “with MACE” (unpaired *t* test, Mann-Whitney *U* test [BNP and Follow-up period] or Chi-squared test [current smoking status, frequent alcohol consumption, proteinuria, hypertension, diabetes mellitus, dyslipidemia, metabolic syndrome]).

^B^Frequent alcohol consumption was defined as the consumption of alcohol six or seven times per week.

Abbreviations: BNP, B-type natriuretic peptide; eGFR, estimated glomerular filtration rate; HDL-C, high-density lipoprotein cholesterol; LDL-C, low-density lipoprotein cholesterol; MACE, major adverse cardiovascular event.

### 3.2 Prospective analysis of cardiovascular risk

**Fig. 2:**
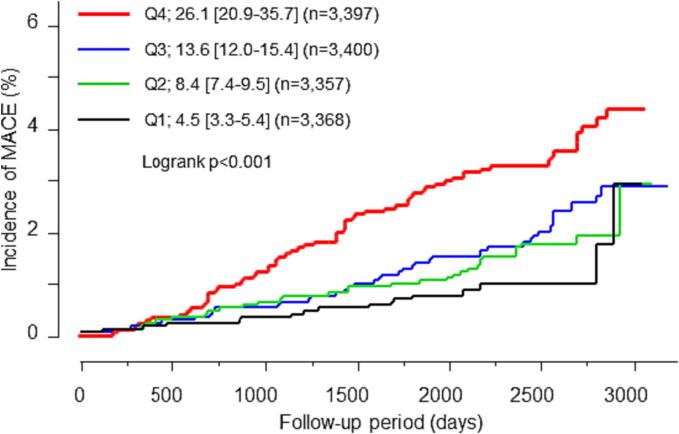
Kaplan-Meier analysis showing the influence of baseline BNP levels on the risk of major adverse cardiovascular events (MACE) in middle-aged study participants, Hamamatsu, Japan, 2008–2016. Participants were divided into four groups according to the quartiles of baseline BNP levels (Q1–4). The median values and interquartile ranges are shown in the figure. Major adverse cardiovascular events are defined as cardiovascular death, non-fatal myocardial infarction, unstable angina, decompensated heart failure, stroke, and other cardiovascular events requiring hospitalization.

Most of the classic risk factors showed a correlation with the risk of MACE, while low-density lipoprotein cholesterol, triglycerides or current smoking habit did not show such correlation in the univariate analysis (Table 4). Among the investigated variables, baseline BNP level was correlated with MACE with the greatest HR. When the participants were divided into four groups according to baseline BNP level quartiles, the risk of MACE was significantly increased across them quartiles (Fig. 2). Factors that showed a significant correlation with MACE after the adjustment for male sex and age (Table 4, Model A) and other important factors were included in a multivariate analysis (Table 4, Model B). The regression analysis showed that male sex, age, waist circumference, systolic blood pressure, and Sokolow-Lyon voltage were independently associated with MACE; however, in a model in which BNP was added as an independent variable, there was no significant correlation between Sokolow-Lyon voltage and MACE, and BNP was the most significant predictor of MACE among the factors included in the model (Table 4, Model C). To confirm the predictive value of BNP, the mean BNP value in the first 2 years after enrollment (baseline, and 1st and 2nd visits thereafter) was calculated and a Cox hazard regression analysis was conducted. The mean BNP was also a significant predictor of MACE after adjustment (HR, 2.300; 95 % CI, 1.306–4.051, *P* = 0.004).

**Table 4:** Cox hazard regression analysis investigating factors that predict the incidence of major adverse cardiovascular events in middle-aged study participants, Hamamatsu, Japan, 2008–2016.

	Univariate		Model A		Model B		Model C	
	*P*	HR (95 % CI)	*P*	HR (95 % CI)	*P*	HR (95 % CI)	*P*	HR (95 % CI)
Sex; male, n (%)	<0.001	2.827 (1.968–4.059)	---	---	0.005	1.811 (1.193–2.746)	0.002	1.973 (1.297–3.000)
Age (years)	<0.001	1.067 (1.053–1.082)	---	---	<0.001	1.061 (1.046–1.077)	<0.001	1.047 (1.031–1.064)
Body mass index (kg/m^2^)	0.007	1.057 (1.015–1.100)	0.009	1.062 (1.015–1.112)	---	---	---	---
Waist circumference (cm)	<0.001	1.034 (1.019–1.049)	0.002	1.027 (1.010–1.044)	0.047	1.018 (1.000–1.037)	0.030	1.020 (1.002–1.038)
Systolic blood pressure (mmHg)	<0.001	1.028 (1.019–1.036)	<0.001	1.017 (1.008–1.025)	0.009	1.012 (1.003–1.022)	0.019	1.011 (1.002–1.020)
Diastolic blood pressure (mmHg)	<0.001	1.032 (1.018–1.046)	0.003	1.022 (1.007–1.037)	---	---	---	---
Pulse rate (bpm)	0.414	0.994 (0.978–1.009)	0.687	1.003 (0.988–1.018)	---	---	---	---
Fasting plasma glucose (mg/dL)	<0.001	1.009 (1.004–1.014)	0.236	1.004 (0.998–1.010)	---	---	---	---
HbA1c (%)	<0.001	1.311 (1.150–1.495)	0.082	1.158 (0.982–1.367)	0.169	1.132 (0.949–1.352)	0.110	1.155 (0.968–1.377)
LDL-C (mg/dL)	0.980	1.000 (0.995–1.005)	0.832	1.001 (0.995–1.006)	0.617	0.999 (0.993–1.004)	0.837	1.001 (0.995–1.006)
HDL-C (mg/dL)	0.002	0.983 (0.972–0.993)	0.126	0.992 (0.981–1.002)	---	---	---	---
Triglyceride (mg/dL)	0.085	1.001 (1.000–1.003)	0.395	1.001 (0.999–1.002)	---	---	---	---
Serum creatinine (mg/dL)	<0.001	1.682 (1.378–2.054)	0.088	1.410 (0.956–2.079)	---	---	---	---
eGFR (mL/min per 1.73 m^2^)	<0.001	0.974 (0.964–0.984)	0.168	0.993 (0.982–1.003)	---	---	---	---
Uric acid (mg/dL)	<0.001	1.273 (1.153–1.405)	0.011	1.164 (1.035–1.308)	0.055	1.124 (0.998–1.266)	0.050	1.126 (1.000–1.268)
Hemoglobin (g/dL)	0.230	1.063 (0.962–1.175)	0.248	0.928 (0.817–1.054)	---	---	---	---
Sokolow-Lyon voltage (mV)	<0.001	1.535 (1.309–1.802)	0.005	1.271 (1.076–1.500)	0.018	1.230 (1.036–1.461)	0.064	1.172 (0.991–1.387)
Current smoking status (%)	0.531	1.111 (0.800–1.543)	0.431	1.150 (0.812–1.627)	0.176	1.276 (0.896–1.818)	0.196	1.262 (0.887–1.797)
Frequent alcohol consumption (%)^A^	0.175	1.212 (0.917–1.613)	0.078	0.758 (0.557–1.032)	---	---	---	---
Proteinuria	0.477	0.603 (0.150–2.429)	0.500	0.619 (0.154–2.496)	---	---	---	---
LogBNP	<0.001	5.068 (3.356–7.656)	<0.001	2.742 (1.727–4.353)	---	---	<0.001	2.662 (1.683–4.209)

All variables included in multivariate analysis are listed in the table.

Model A: Impact of each variable on the development of MACE was analyzed after adjustment for male sex and age.

Model B: Male sex, age, waist circumference, systolic blood pressure, HbA1c, LDL-C, uric acid, Sokolow-Lyon voltage, and current smoking status were included in the multivariate model as independent variables.

Model C: LogBNP was additionally included in the multivariate model B.

^A^Frequent alcohol consumption was defined as the consumption of alcohol six or seven times per week.

Abbreviations: BNP, B-type natriuretic peptide; eGFR, estimated glomerular filtration rate; HbA1c, hemoglobin A1c; HDL-C, high-density lipoprotein cholesterol; LDL-C, low-density lipoprotein cholesterol.

### 3.3 Risk of hypertension, diabetes mellitus, dyslipidemia, and metabolic syndrome

**Fig. 3:**
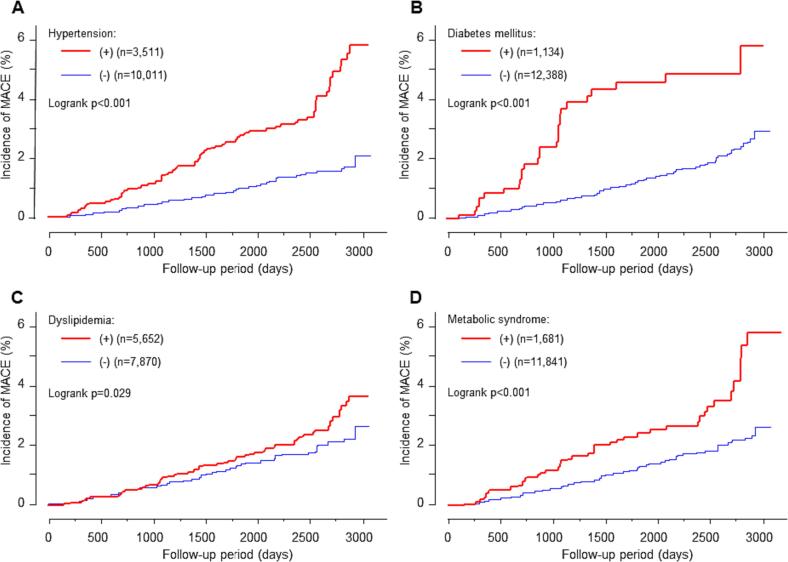
Kaplan-Meier analysis showing the influence of hypertension (A), diabetes mellitus (B), dyslipidemia (C), and metabolic syndrome (D) at baseline on the risk of major adverse cardiovascular events (MACE) in middle-aged study participants, Hamamatsu, Japan, 2008–2016. Major adverse cardiovascular events are defined as cardiovascular death, non-fatal myocardial infarction, unstable angina, decompensated heart failure, stroke, and other cardiovascular events requiring hospitalization.

Another series of analyses investigated the predictive value of hypertension, diabetes mellitus, dyslipidemia, and metabolic syndrome for MACE (Fig. 3, Table 5). Although participants with such diseases were at increased risk of MACE in the Kaplan-Meier analysis (Fig. 3) and univariate analysis (Table 5), hypertension, diabetes mellitus, and metabolic syndrome, but not dyslipidemia, were independent predictors of MACE according to the multivariate Cox hazard analysis (Table 5).

**Table 5:** Cox hazard regression analysis investigating the predictive value of lifestyle-related diseases for major adverse cardiovascular events in middle-aged study participants, Hamamatsu, Japan, 2008–2016.

	Univariate		Model A		Model B		Model C	
	*P*	HR (95 % CI)	*P*	HR (95 % CI)	*P*	HR (95 % CI)	*P*	HR (95 % CI)
^a^Hypertension (%)	<0.001	2.809 (2.121–3.720)	<0.001	1.809 (1.350–2.425)	0.006	1.543 (1.134–2.099)	0.010	1.506 (1.105–2.053)
^b^Diabetes mellitus (%)	<0.001	2.989 (2.003–4.461)	0.001	1.962 (1.310–2.939)	0.002	1.902 (1.262–2.866)	0.002	1.930 (1.280–2.910)
^c^Dyslipidemia (%)	0.029	1.367 (1.032–1.812)	0.145	1.233 (0.930–1.634)	0.746	1.049 (0.783–1.406)	0.340	1.156 (0.859–1.554)
^d^Metabolic syndrome (%)	<0.001	2.032 (1.456–2.836)	0.045	1.414 (1.008–1.982)	0.066	1.376 (0.979–1.933)	0.036	1.439 (1.024–2.023)

Model A: Impact of lifestyle-related diseases on the development of MACE was analyzed after adjustment for male sex and age.

Model B:

^a^Predictive value of hypertension was analyzed after adjustment for male sex, age, waist circumference, HbA1c, LDL-C, uric acid, Sokolow-Lyon voltage, and current smoking status.

^b^Predictive value of diabetes mellitus was analyzed after adjustment for male sex, age, waist circumference, systolic blood pressure, LDL-C, uric acid, Sokolow-Lyon voltage, and current smoking status.

^c^Predictive value of dyslipidemia was analyzed after adjustment for male sex, age, waist circumference, systolic blood pressure, HbA1c, uric acid, Sokolow-Lyon voltage, and current smoking status.

^d^Predictive value of metabolic syndrome was analyzed after adjustment for male sex, age, uric acid, Sokolow-Lyon voltage, and current smoking status.

Model C was additionally adjusted for logBNP.

Abbreviations: BNP, B-type natriuretic peptide; eGFR, estimated glomerular filtration rate; HbA1c, hemoglobin A1c; HDL-C, high-density lipoprotein cholesterol; LDL-C, low-density lipoprotein cholesterol.

### 3.4 Sub-analysis of participants without medication

In a univariate sub-analysis of participants who were not taking medication that could affect the cardiovascular system (*n* = 10,475), male sex, age, blood pressure, serum creatinine level, eGFR, uric acid level, Sokolow-Lyon voltage, proteinuria, and BNP were significantly associated with MACE (Table 6). However, male sex, age, blood pressure, and BNP were the only independent predictors of MACE (Table 6, Model C).

**Table 6:** Cox hazard regression analysis investigating factors that predict the incidence of major adverse cardiovascular events in middle-aged study participants without medication, Hamamatsu, Japan, 2008–2016.

	Univariate		Model A		Model B		Model C	
	*P*	HR (95 % CI)	*P*	HR (95 % CI)	*P*	HR (95 % CI)	*P*	HR (95 % CI)
Sex; male, n (%)	<0.001	2.680 (1.643–4.373)			0.004	2.160 (1.288–3.623)	0.001	2.410 (1.428–4.067)
Age (years)	<0.001	1.070 (1.050–1.090)			<0.001	1.065 (1.044–1.085)	<0.001	1.050 (1.027–1.073)
Body mass index (kg/m^2^)	0.339	1.030 (0.969–1.095)	0.320	1.035 (0.967–1.108)	---	---	---	---
Waist circumference (cm)	0.075	1.020 (0.998–1.043)	0.335	1.012 (0.988–1.037)	---	---	---	---
Systolic blood pressure (mmHg)	<0.001	1.029 (1.017–1.041)	0.004	1.018 (1.006–1.031)	0.018	1.016 (1.003–1.029)	0.024	1.015 (1.002–1.028)
Diastolic blood pressure (mmHg)	<0.001	1.044 (1.024–1.064)	0.001	1.035 (1.014–1.056)	---	---	---	---
Pulse rate (bpm)	0.367	0.990 (0.968–1.012)	0.858	1.002 (0.981–1.024)	---	---	---	---
Fasting plasma glucose (mg/dL)	0.641	1.003 (0.991–1.015)	0.331	0.992 (0.976–1.008)	---	---	---	---
HbA1c (%)	0.482	1.116 (0.822–1.515)	0.359	0.808 (0.513–1.274)	0.253	0.763 (0.479–1.213)	0.297	0.782 (0.492–1.242)
LDL-C (mg/dL)	0.207	1.004 (0.998–1.011)	0.368	1.003 (0.996–1.010)	0.409	1.003 (0.996–1.010)	0.171	1.005 (0.998–1.013)
HDL-C (mg/dL)	0.375	0.994 (0.980–1.008)	0.774	1.002 (0.988–1.016)	---	---	---	---
Triglyceride (mg/dL)	0.607	1.001 (0.998–1.003)	0.806	1.000 (0.997–1.002)	---	---	---	---
Serum creatinine (mg/dL)	<0.001	4.541 (1.961–10.51)	0.601	1.461 (0.353–6.051)	---	---	---	---
eGFR (mL/min per 1.73 m^2^)	0.002	0.977 (0.962–0.992)	0.623	0.996 (0.981–1.011)	---	---	---	---
Uric acid (mg/dL)	0.003	1.229 (1.072–1.408)	0.167	1.124 (0.952–1.327)	---	---	---	---
Hemoglobin (g/dL)	0.709	1.026 (0.897–1.173)	0.087	0.859 (0.723–1.022)	---	---	---	---
Sokolow-Lyon voltage (mV)	<0.001	1.649 (1.319–2.061)	0.006	1.384 (1.096–1.748)	0.030	1.305 (1.026–1.661)	0.072	1.244 (0.981–1.578)
Current smoking status (%)	0.849	0.956 (0.605–1.512)	0.686	0.906 (0.561–1.463)	0.905	1.030 (0.633–1.677)	0.884	1.037 (0.637–1.688)
Frequent alcohol consumption (%)^A^	0.068	1.439 (0.974–2.126)	0.721	0.926 (0.606–1.414)	---	---	---	---
Proteinuria	0.021	0.462 (0.240–0.889)	0.111	0.587 (0.305–1.129)	---	---	---	---
LogBNP	<0.001	5.336 (2.925–9.732)	0.002	2.828 (1.445–5.534)	---	---	0.004	2.724 (1.373–5.405)

All variables included in multivariate analysis are listed in the table.

Model A: Impact of each variable on the development of MACE was analyzed after adjustment for male sex and age.

Model B: Male sex, age, systolic blood pressure, HbA1c, LDL-C, Sokolow-Lyon voltage, and current smoking status were included in the multivariate model as independent variables.

Model C: LogBNP was additionally included in the multivariate model B.

^A^Frequent alcohol consumption was defined as the consumption of alcohol six or seven times per week.

Abbreviations: BNP, B-type natriuretic peptide; eGFR, estimated glomerular filtration rate; HbA1c, hemoglobin A1c; HDL-C, high-density lipoprotein cholesterol; LDL-C, low-density lipoprotein cholesterol.

## 4 Discussion

The present study demonstrated that plasma BNP level obtained at medical checkups of the general Japanese population is a strong independent predictor of MACE. Thus, the addition of BNP to the medical checkup parameters may improve the quality of cardiovascular disease predictions and, therefore, play a role in cardiovascular disease prevention.

BNP is synthesized by and secreted from ventricular cells, mainly in response to an increase in ventricular volume or pressure (Mukoyama et al., 1991, Hirata et al., 2001, Maries and Manitiu, 2013, Nakagawa et al., 2019, Goetze et al., 2020). The peptide exerts a protective action against cardiac load or damage. Indeed, BNP has a direct antifibrotic effect in the heart that prevents cardiac remodeling (Kapoun et al., 2004). There is increasing evidence that circulating plasma BNP level is a reliable marker of left ventricular function and heart failure severity and prognosis (Di Angelantonio et al., 2009; Maries et al., 2013; Nakagawa et al., 2019, Goetze et al., 2020). Furthermore, BNP-guided treatment is effective in patients at high risk, such as those with diabetes mellitus, to reduce left ventricular dysfunction or cardiovascular events (Huelsmann et al., 2013; Ledwidgge et al., 2013; Sweeney et al., 2019). However, careful interpretation is necessary because numerous factors, such as sex, age, blood pressure, kidney function, and obesity, seriously affect BNP levels (Maries et al., 2013; Madamanchi et al., 2014, Nakagawa et al., 2019, Goetze et al., 2020, Ohashi et al., 2022).

Moreover, the clinical significance of a relatively low BNP level seems obscure, and a more detailed investigation is necessary to clarify whether BNP level can be useful in the general population (Di Angelantonio et al., 2009). Although the normal reference BNP level is ≤ 18.4 pg/mL, a value above normal does not necessarily indicate heart failure. Relatively low BNP levels predict an increased risk of left ventricular dysfunction or heart failure in patients with diabetes, while the threshold of BNP was ≥ 50 pg/mL and that of N-terminal prohormone BNP was ≥ 125 pg/mL (Ledwidge et al., 2013, Pop-Busui et al., 2022). The Japanese Heart Failure Society set a cut-off point of 35 pg/mL for plasma BNP to identify patients with mild heart failure and a cut-off of 100 pg/mL to identify patients with heart failure requiring medication (Preventive Committee for Japanese Heart Failure Society, 2023). A BNP < 35 pg/mL has been considered low for identifying heart failure. In the present study, the median value and interquartile range of BNP was 10.7 and 6.4–17.8 pg/mL, respectively. Thus, the present results clearly demonstrate the clinical significance of relatively low BNP levels in the general population and the beneficial effect of BNP measurement in medical checkups. The present results are consistent with that of a previous community-based study in which 3,346 participants had a median BNP of 6.2 pg/mL for men and 10.0 pg/mL for women (Wang et al., 2004). A total of 79 cardiovascular events occurred during a mean of 5.2 years of follow-up, and increasing BNP levels for 1 SD in log values were associated with an 28 % elevated risk of MACE (Wang et al., 2004). It is noteworthy that BNP measured during medical checkup predicts MACE independent of classic risk factors, such as hypertension, diabetes mellitus, dyslipidemia, and smoking. This present finding implies that BNP and classic risk factors play a complementary role in the prediction and subsequent prevention of MACE. The results of the sub-analysis of participants not taking medication that may affect the cardiovascular system further support the concept that BNP is a strong independent predictor of MACE. However, careful interpretation is necessary because more than a half of the participants with hypertension, diabetes mellitus, or dyslipidemia were excluded from the sub-analysis, and blood pressure, glucose tolerance, and lipid values were nearly normal in most of the participants included in the sub-analysis.

The mechanism underlying the association between BNP and the future occurrence of MACE was not elucidated in the present study. The prediction of heart failure using BNP levels is easy to understand when considering the mechanism of BNP secretion (Mukoyama et al., 1991, Hirata et al., 2001, Maries and Manitiu, 2013, Nakagawa et al., 2019, Goetze et al., 2020). Latent left ventricular dysfunction induced by subclinical or concealed myocardial ischemia may have increased the BNP levels, resulting in its prediction of clinical myocardial ischemia, such as myocardial infarction and angina requiring hospitalization. In line with this speculation, BNP level predicts silent myocardial ischemia in patients with non-obstructive hypertrophic cardiomyopathy (Nakamura et al., 2002) and predicts the recurrence of angina pectoris (Takase et al., 2004). Recent studies suggested that salt intake causes a mild BNP elevation (<30 pg/mL) in the general population (Ohashi et al., 2021, Ohashi et al., 2022) and that an excessive salt intake is an independent risk factor for cardiovascular events (Aburto et al., 2013). Thus, the mild increase in BNP observed in the present study may at least partially reflect an excessive salt intake. BNP levels are also elevated in patients with hypertension, and an increased blood pressure stimulates BNP secretion (Jakubik et al., 2006, Dohi, 2022 Jul), suggesting that an increased BNP level may have partially occurred following a mild blood pressure elevation, although the adjustment for blood pressure in the Cox regression analysis did not eliminate the significant correlation between BNP and MACE.

The present study confirmed the importance of the identification of individuals with metabolic syndrome as well as other classic risk factors. Although waist circumference, high-density lipoprotein cholesterol level, or triglyceride level alone was not an independent risk factor for MACE in the present observational study, a cluster of mild metabolic disorders independently predicted future cardiovascular events. The reason why low-density lipoprotein cholesterol did not show a significant correlation with MACE is not clear; however, this may be at least partially attributable to the relatively low levels of low-density lipoprotein cholesterol in the present study participants (120.4 ± 28.7 mg/dL). The inclusion of ex-smokers may have affected the result that smoking was not a cardiovascular risk, although the detailed mechanisms of this phenomenon are uncertain.

The interpretation of the present results is limited by the following points. All study subjects were participants in our annual medical checkup program; thus, selection bias is possible. In some cases, the outcome was confirmed using a questionnaire followed by a detailed history taking and medical examination by a doctor at medical checkup or by a letter, leading to a likelihood of an inaccurate diagnosis of the outcome in those participants. Some participants dropped out (dropout rate = 6.7 %), which may have affected the results. Information about past smoking or drinking status was not available. Although the large number of participants included in the present study may overcome some of these limitations, a study with more participants and longer observation period is necessary to arrive at a definite conclusion. These points should be noted when interpreting the present data.

## 5 Conclusion

Plasma BNP levels measured during medical checkups could serve as an excellent predictor of MACE. The inclusion of BNP as part of medical checkup parameters may improve the predictive quality of medical checkups in the general Japanese population.

## Funding

This work was partially supported by JSPS KAKENHI (Grant Number JP 20 K07854).

## CRediT authorship contribution statement

**Tomonori Sugiura:** Writing – review & editing, Writing – original draft, Methodology, Conceptualization. **Hiroyuki Takase:** . **Yasuaki Dohi:** Writing – review & editing, Methodology, Conceptualization. **Sumiyo Yamashita:** Writing – review & editing. **Yoshihiro Seo:** Conceptualization.

## Declaration of competing interest

The authors declare that they have no known competing financial interests or personal relationships that could have appeared to influence the work reported in this paper.

## Data Availability

No data was used for the research described in the article.
